# Antimicrobial resistance of *Pasteurella multocida* type B isolates associated with acute septicemia in pigs and cattle in Spain

**DOI:** 10.1186/s12917-020-02442-z

**Published:** 2020-06-30

**Authors:** Inmaculada Cuevas, Alfonso Carbonero, David Cano, Ignacio García-Bocanegra, Manuel Ángel Amaro, Carmen Borge

**Affiliations:** 1 Department of Animal Health (AGR-149). Campus of International Agri-Food Excellence CeiA3, University of Córdoba, Rabanales University Campus, 14071 Córdoba, Spain; 2 Department of Bromatology. Campus of International Agri-Food Excellence CeiA3, University of Córdoba, Rabanales University Campus, 14071 Córdoba, Spain

**Keywords:** *Pasteurella multocida*, Type B, Antimicrobial susceptibility, Resistance, Hemorrhagic septicemia

## Abstract

**Background:**

*Pasteurella multocida* is the etiological agent responsible for several diseases in a wide range of hosts around the world and thus, causes serious economic losses. Acute septicemia associated with capsular type B *P. multocida* has recently emerged in Europe and continuous outbreaks of these acute processes have been described in Spain since they were first detected in pigs in 2009 and cattle in 2015. The scarcity of studies on the antimicrobial susceptibility of this capsular type of *P. multocida* and growing concern about the general increase of antimicrobial resistance mean that studies related to the performance of type B *P. multocida* against antibiotics are necessary to establish accurate treatments and to monitor antimicrobial resistances.

**Results:**

Seventy-six isolates of *P. multocida* type B from pigs and cattle with acute septicemia were tested for susceptibility to 10 different antimicrobials. Bovine isolates were susceptible to all the antibiotics we tested except for lincomycin (94.4% of isolates were resistant). However, the antimicrobials we tested were less effective against swine isolates, of which none were susceptible to lincomycin. Furthermore, 29.3% swine isolates were resistant to tetracycline, 27.6% to penicillin, 20.7% to oxytetracycline, 17.3% to chloramphenicol, 15.5% to gentamicin, and 3.4% to enrofloxacin; no resistance to ceftiofur was detected. No multidrug resistant isolates were detected from cattle, while 25.86% of swine isolates were resistant to three or more antibiotic classes.

**Conclusions:**

In this study, the lower resistance rates and multidrug resistant isolates reported for *P. multocida* type B derived from cattle compared to those isolated from pigs may be related to the increased use of antibiotics in the porcine industry in Spain. Lincomycin is not recommended for the treatment of acute septicemia in pigs or cattle, rather, the use of ceftiofur, enrofloxacin, or gentamicin is indicated as an emergency treatment in the early stages of disease; once the susceptibility results are known, the use of tetracyclines, penicillin, or chloramphenicol should be prioritized. The increase in multidrug resistant isolates and antimicrobial resistance rates indicates that more attention should be paid to prevention as well as the responsible use of antibiotics.

## Background

Even though *Pasteurella multocida* is commonly present in the upper respiratory tract of domestic and wild species and it is considered an opportunistic agent, this microorganism is, in fact, the primary etiological cause of a wide range of diseases with global economic importance [1]. *P. multocida* affects a broad range of hosts and causes many diseases including pneumonia in pigs, cattle, small ruminants, and rabbits, acute septicemia in cattle and buffalo, atrophic rhinitis in swine, and fowl cholera in poultry [2–5].

The acute septicemia caused by *P. multocida* is associated with hemorrhagic septicemia (HS), a severe disease which involves the capsular types B and E of this microorganism [4]. HS is endemic in some areas of Asia and Africa and commonly affects cattle and buffalo, but is also infrequently reported in pigs [6]. However, only sporadic outbreaks of HS affecting pigs have been described in limited geographic areas including India [7], Sri Lanka [8], Vietnam [6], and Australia [9].

Capsular type B *P. multocida* causing acute septicemia has recently emerged in Europe. The first outbreak of HS caused by this agent was reported in 2009 in Spain and affected Iberian pigs from seven herds reared in an extensive farm system [10]. Since then, several authors have described periodic outbreaks of acute septicemia in Iberian pigs [11], wild boar [12] and cattle [13] in Spain. Additionally, sporadic outbreaks of HS in pigs and cattle have also recently been reported in Hungary [14, 15].

Antimicrobial therapy is still proven to be effective in the treatment of diseases caused by *P. multocida*, including HS. The etiological treatment of this disease is based on the parenteral administration of antibiotics (e.g., penicillin, ampicillin, tetracycline, chloramphenicol, streptomycin, or neomycin), which are only effective in affected animals in the early stages of disease [4, 16]. However, the excessive and unreasonable use of antimicrobials has accelerated the selective pressure put on the expression of genes encoding resistance in these microorganisms thus, increasing the emergence of resistant isolates [17]. Indeed, an increase in the incidence of multidrug resistant (MDR) pathogenic bacteria has been reported in recent decades [18] and is decreasing the efficacy of currently available antibiotics used to treat infectious diseases in food-producing animals.

Moreover, antimicrobial resistances impact public health because of the potential for zoonotic bacteria to pass onto humans through the food chain [19]. The growing concern about the increase in antimicrobial resistances has meant that national and international action has been taken to monitor, harmonize, and prudently use antibiotics [20, 21]. The World Organisation for Animal Health (OIE) and World Health Organisation (WHO) have each developed a list of critically important antimicrobial agents in veterinary medicine and human medicine, respectively [22, 23].

The proper use of antibiotics to control pasteurellosis will require the completion of detailed surveys in several geographical areas [17]. Furthermore, because of the recent emergence and circulation of *P. multocida* type B in Europe and the lack of studies regarding the response of this capsular type to antibiotics, its antimicrobial susceptibility profile must be determined in order to establish accurate treatments and to detect and monitor antibiotic resistances.

## Results

Seventy-six isolates from swine (*n =* 58) and cattle (*n =* 18) affected with acute septicemic pasteurellosis caused by *P. multocida* type B, biovar 3 were tested for susceptibility to 10 different antimicrobial agents. The percentage of isolates which were susceptible, intermediate, or resistant to each antibiotic are listed in Table 1 and their minimum inhibitory concentration (MIC) distribution and MIC_50_ and MIC_90_ are shown in Tables 2 and 3.

**Table 1:** Antibiotic susceptibility frequencies of 76 *Pasteurella multocida* type B isolates

Antimicrobial agent	Susceptibility frequencies (%) to individual antibiotic								
	Swine (*n =* 58)			Cattle (*n =* 18)			Total (*n =* 76)		
	S	I	R	S	I	R	S	I	R
**ENR**	86.2	6.9	6.9	94.4	5.6	0	88.2	6.6	5.3
**CEF**	98.3	1.7	0	100	0	0	98.7	1.3	0
**GEN**	84.5	0	15.5	100	0	0	88.2	0	11.8
**C**	81.0	1.7	17.3	100	0	0	85.5	1.3	13.2
**LIN**	0	0	100	5.6	0	94.4	1.3	0	98.7
**PEN**	72.4	0	27.6	72.2	22.2	5.6	72.4	5.3	22.4
**OXY**	77.6	1.7	20.7	100	0	0	82.9	1.3	15.8
**TE**	58.6	12.1	29.3	94.4	5.6	0	67.1	10.5	22.4
**ERY**	ND	ND	ND	ND	ND	ND	ND	ND	ND
**NEO**	ND	ND	ND	ND	ND	ND	ND	ND	ND

*C* Chloramphenicol; *CEF* Ceftiofur; *ENR* Enrofloxacin; *ERY* Erythromycin; *GEN* Gentamicin; *I* Intermediate; LIN Lincomycin; *ND* Non-determined value; *NEO* Neomycin; *OXY* Oxytetracycline; *PEN* Penicillin; *S* Susceptible; *R* Resistant; *TE* Tetracycline.

**Table 2:** MIC value distribution of *Pasteurella multocida* type B isolates derived from swine (*n =* 58)

Break point	Ab	Number of isolates for each MIC (μg/mL)										MIC_50_	MIC_90_	%R
		≤0.03	0.06	0.125	0.25	0.5	1	2	4	8	≥16			
≥1	**ENR**	45	2	2	1	4	3	0	1	0	0	≤0.03	0.5	6.9
≥8	**CEF**	39	3	0	1	4	7	3	1	0	0	≤0.03	1	0
> 8	**GEN**	0	0	0	0	0	3	11	16	19	9	4	≥16	15.5
≥32	**C**	0	0	0	2	29	6	3	3	4	>11^c^	0.5	≥16	17.2
> 2	**LIN**	0	0	0	0	0	0	0	0	6	52	≥16	≥16	100
≥1	**PEN**	10	12	17	3	0	0	16	0	0	0	0.125	2	27.6
≥2^a^	**OXY**	4	3	6	26	6	1	2	1	9	0	0.25	8	20.7
≥2	**TE**	3	0	6	16	9	7	7	3	7	0	0.5	8	29.3
ND	**ERY**	0	0	2	2	3	8	1	8	0	34	≥16	≥16	ND
ND^b^	**NEO**	0	0	0	0	0	1	6	14	20	17	8	≥16	ND

^a^ breakpoint reference value for tetracycline; ^b^ breakpoint value for *P. multocida* derived from dogs; ^c^ MIC value = 32 μg/mL. *Ab* Antibiotic; *C* Chloramphenicol; *CEF* Ceftiofur; *ENR* Enrofloxacin; *ERY* Erythromycin; *GEN* Gentamicin; *LIN* Lincomycin; *MIC* Minimum inhibitory concentration; *ND* Non-determined value for *P. multocida* according to the Clinical and Laboratory Standards Institute (CLSI; 2013); *NEO* Neomycin; *OXY* Oxytetracycline; *PEN* Penicillin; *TE* Tetracycline

**Table 3:** MIC value distribution of *Pasteurella multocida* type B isolates derived from cattle (*n =* 18)

Break point	Ab	Number of isolates for each MIC (μg/mL)										MIC_50_	MIC_90_	%R
		≤0.03	0.06	0.125	0.25	0.5	1	2	4	8	≥16			
≥1	**ENR**	16	1	0	0	1	0	0	0	0	0	≤0.03	0.06	0
≥8	**CEF**	16	0	1	0	0	1	0	0	0	0	≤0.03	0.125	0
> 8	**GEN**	1	0	0	1	0	0	10	6	0	0	2	4	0
≥32	**C**	0	0	0	0	17	1	0	0	0	0	0.5	0.5	0
> 2	**LIN**	0	0	0	0	0	0	1	0	6	11	≥16	≥16	94.4
≥1	**PEN**	1	10	1	1	4	1	0	0	0	0	0.06	0.5	5.6
≥8	**OXY**	2	2	1	10	3	0	0	0	0	0	0.25	0.5	0
≥8	**TE**	1	0	2	6	1	5	2	1	0	0	0.25	2,0	0
ND	**ERY**	0	0	1	6	3	0	0	0	0	8	0.5	≥16	ND
ND	**NEO**	0	1	0	0	1	0	0	14	2	0	0.4	8	ND

^a^ breakpoint reference value for tetracycline; ^b^ breakpoint value for *P. multocida* derived from dogs. *Ab* Antibiotic; *C* Chloramphenicol; *CEF* Ceftiofur; *ENR* Enrofloxacin; *ERY* Erythromycin; *GEN* Gentamicin; *LIN* Lincomycin; *MIC* Minimum inhibitory concentration; *ND* Non-determined value for *P. multocida* according to the Clinical and Laboratory Standards Institute (CLSI; 2013); *NEO* Neomycin; *OXY* Oxytetracycline; *PEN* Penicillin; *TE* Tetracycline

Most isolates were susceptible to the antimicrobial agents tested, except for lincomycin: 100% of porcine isolates and 94.4% of bovine isolates were resistant to this drug. There was no evidence of any resistance to enrofloxacin, ceftiofur, gentamicin, chloramphenicol, oxytetracycline, or tetracycline in bovine isolates. Bacteria isolated from cattle only showed a high percentage of resistance to lincomycin, with low levels of resistance to penicillin (5.6%). Regarding the porcine isolates, 29.3% were resistant to tetracycline, 27.6% to penicillin, 20.7% to oxytetracycline, 17.3% to chloramphenicol, and 15.5% to gentamicin. A small number of isolates were resistant to enrofloxacin (6.9%) while no resistance to ceftiofur was detected. The resistance rates of penicillin (*p* = 0.05) and oxytetracycline (*p* = 0.035) were significantly higher in swine than in cattle. There was no statistical difference in the rest of antimicrobial agents tested between both host species.

The resistance rates to erythromycin and neomycin could not be determined in any of the isolates because the break points of these drugs for *P. multocida* isolates have not yet been defined in the Clinical and Laboratory Standards Institute (CLSI) criteria. The MIC values for porcine and bovine isolates ranged between 0.125 and ≥ 16 μg/mL for erythromycin, and most swine isolates (34/58) had a MIC value ≥16 μg/mL. For neomycin, the MIC values of porcine isolates were between 1 and ≥ 16 μg/mL and were from 0.06 to 8 μg/mL for bovine isolates. Interestingly, swine and bovine isolates had the same MIC_50_ values for enrofloxacin (≤0.03 μg/mL), ceftiofur (≤0.03 μg/mL), chloramphenicol (0.5 μg/mL), lincomycin (≥16 μg/mL), and oxytetracycline (0.25 μg/mL). However, the MIC_50_ was higher in swine isolates than in bovine isolates for gentamicin, penicillin, erythromycin, and neomycin. More marked differences were found in the MIC_90_ values, which were higher for the porcine isolates than for the bovine ones, except for lincomycin and erythromycin (≥16 μg/mL in both hosts). The distribution of the antimicrobial susceptibility patterns for *P. multocida* type B isolates from swine and cattle are summarized in Table 4.

**Table 4:** Patterns of antimicrobial resistance of *P. multocida* isolates from swine and cattle

Patterns of antimicrobial resistance	Antibiotic classes (*n*)	Porcine isolates (*n*)	Bovine isolates (*n*)	Total Isolates (*n*)
LIN	1	37	14	51
PEN	1	0	1	1
C-LIN	2	1	0	1
ENR-LIN	2	1	0	1
LIN-PEN	2	1	0	1
LIN-TE	2	3	3	6
GEN-LIN-PEN	3	1	0	1
LIN-PEN-TE	3	2	0	2
LIN-PEN-OXY-TE^a^	3	2	0	2
C-LIN-PEN-OXY-TE^a^	4	2	0	2
GEN-LIN-PEN-OXY-TE^a^	4	1	0	1
GEN-C-LIN-PEN-OXY-TE^a^	5	6	0	6
ENR-GEN-C-LIN-PEN-OXY-TE^a^	6	1	0	1

^a^ OXY-TE: OXY and TE belong to the same group of antibiotics. *C* Chloramphenicol; *CEF* Ceftiofur; *ENR* Enrofloxacin; *ERY* Erythromycin; *GEN* Gentamicin; *LIN* Lincomycin; *NEO* Neomycin; *OXY* Oxytetracycline; *PEN* Penicillin; *TE* Tetracycline

Of note, all the isolates from both species were resistant at least to one of the 10 drugs we tested. While no MDR strains were found for the bovine isolates, 25.86% of the porcine isolates were multiresistant (*p* = 0.016). Importantly, lincomycin and penicillin resistance were present in all the MDR patterns we identified.

## Discussion

To the best of our knowledge, this study represents the first research performed in Europe about the antimicrobial susceptibility of capsular type B *P. multocida* derived from clinical samples from pigs and cattle affected with acute septicemia. However, the antimicrobial susceptibility of capsular types A and D from porcine isolates have been previously discussed for Spain [24, 25], China [18], Brazil [26], and Korea [27]. In addition, other authors have studied antimicrobial resistance rates in *P. multocida* isolates from cattle in Europe [28] and the United States [29]. All of these previous data are summarized in Table 5.

**Table 5:** Resistance rates found in other publications compared to the results of this current study

Ab	Lizarazo et al. [24]		Petrocchi-R et al. [25]	Tang et al. [18]	Furian et al. [26]	El Garch et al. [28]	Timsit et al. [29]	Oh et al. [27]	This study	
	1987–1988	2003–2004							Swine	Bovine
**ENR**	0	0	0	–	22.5	0	0	2.6	6.9	0
**CEF**	0	0	0	0	22.5	0	0	0.2	0	0
**GEN**	0	0	–	13.7	2.5	–	–	3.3	15.5	0
**C**	1.6	0.7	0	2.6	–	–	–	–	17.2	0
**LIN**	–	–	–	96.6	–	100	–	–	100	94.4
**PEN**	1.6	3.8	–	–	–	–	0	5.5	27.6	5.6
**AMP**	1.6	3	40.6	–	–	–	–	4.8	–	–
**OXY**	1.6	14.4	–	–	–	–	–	66.5	20.7	0
**TE**	–	–	18.8	58	40	20.4	83	–	29.3	0
**ERY**	1.6	3.8	12.5	6	40	–	–	–	ND	ND
**NEO**	1.6	0	–	ND	–	–	–	ND	ND	ND

*Ab* Antibiotic; *C* Chloramphenicol; *CEF* Ceftiofur; *ENR* Enrofloxacin; *ERY* Erythromycin; *GEN* Gentamicin; *LIN* Lincomycin; *ND* Non-determined value; *NEO* Neomycin; *OXY* Oxytetracycline; *PEN* Penicillin; *TE* Tetracycline; − = antimicrobial agent not tested by these authors.

The sale of veterinary antibiotics in Spain is among the highest in Europe [30]. To promote the prudent use of antibiotics, antimicrobial resistances must first be detected and monitored. This data must then be made available to veterinarians so that they can implement effective therapies. Furthermore, it is important to define the antimicrobial susceptibility profile of type B *P. multocida* so that acute pasteurellosis can be treated as early as possible with effective antibiotics.

In our study, 18 *P. multocida* type B isolates from diseased cattle were highly susceptible to all the antibiotics we tested, except for lincomycin (94.4% of the isolates were resistant). The MIC values for this antimicrobial agent varied from 2 to ≥16 μg/mL and the MIC_50_ and MIC_90_ were both ≥16 μg/mL. These values exceeded the break point (> 2 μg/mL), indicating the presence of antimicrobial resistance to this drug. Compared to our work, other authors who studied *P. multocida* isolated from cases of pneumonia from across Europe [28], found higher MIC values (between 16 and 64 μg/mL) in bovine isolates, with the MIC for most isolates being 32 μg/mL.

Timsit et al. [29] also described higher resistance rates in *P. multocida* isolates from a Canadian feedlot (83% of these isolates were resistant to oxytetracycline), both from healthy cattle and those affected with bovine respiratory disease. However, oxytetracycline and other antibiotics were used at this feedlot before the enrollment, which may help to explain these results. Even so, in agreement with our results, these authors found no resistances to ceftiofur or enrofloxacin. The absence or low levels of antibiotic resistances we found for type B bovine *P. multocida* in this study might be expected because this pathogen has only recently emerged in cattle in Spain [13], meaning that this type have been subjected to very little selective pressure to date. In addition, so far, *P. multocida* type B has only been detected in animals reared in extensive systems.

The same as our findings in cattle, lincomycin was completely ineffective against type B *P. multocida* derived from porcine isolates. The MIC values of this drug varied from 8 to ≥16 μg/mL in pigs and the MIC_50_ and MIC_90_ were both ≥16 μg/mL. Lincomycin resistance has previously been reported in *P. multocida* type A or D isolates from pigs in China [18] with 96.6% of isolates resistant to this antibiotic and an MIC_50_ of 8 μg/mL and MIC_90_ of 32 μg/mL reported. Lincomycin has not yet been tested in this context in Spain, although some authors have researched resistance to clindamycin, an antibiotic from the same family of antimicrobials. Petrocchi-Rilo et al. [25] found 96.9% resistance (31/32 isolates) to clindamycin, while El Garch et al. [28] described in Europe MIC values to lincomycin between 4 and 64 μg/mL in porcine isolates (the mode value was 32 μg/mL). According to the break point specified by the Clinical and Laboratory Standards Institute (CLSI) for lincomycin, all isolates from El Garch et al. [28] would have been classified as resistant. The low activity of lincosamides against Pasteurellaceae is well known [31] and the selection of this antimicrobial in the study reflects in the number of MDR patterns identified. According to our data and these previously published results, the use of antimicrobials in the lincosamides family is unlikely to effectively treat cattle or pigs affected with *P. multocida* and thus, is not recommended.

In Spain, the rate of resistance to tetracyclines did not excessively increase from 2004 (14.4%) to 2018 (18.8%) for type A or D *P. multocida* isolates from pigs [25]. Here, we found that 29.3 and 20.7% of *P. multocida* type B isolates from pigs were resistant to tetracycline and oxytetracycline, respectively. According to El Garch et al., the resistance rate of *P. multocida* isolates to tetracycline in Europe and Australia (between 11.5 and 32.2%) were the highest from among several antimicrobials tested [28]. Higher resistance values have been obtained for type A or D *P. multocida* from swine for tetracycline in China (58% [18]; and oxytetracycline in Korea (66.5% [27];, which may be related to the heavy use of antimicrobials in these countries compared to the European Union, especially in the pig industry [27]. Furian et al. [26] also found high resistance to tetracycline in Brazil with 40% of the type A or D *P. multocida* isolates they had derived from pigs being resistant to this drug.

ß-lactams are widely used in the treatment and prevention of swine respiratory tract diseases. This group of antibiotics showed high efficacy (> 96% susceptibility) against *P. multocida* type A or D from swine 15 years ago in Spain [24]. However, their wide use has exerted selective resistance pressure on pathogens so that *P. multocida* isolated from pigs in Spain currently show resistance to penicillin and ampicillin in 27.6% (in this study) and 40.6% [25] of cases, respectively. The percentage of *P. multocida* type B isolates resistant to penicillin may be lower because acute septicemia has appeared in Spain relatively recently. In contrast, ß-lactams remain effective in Korea where, according to one report, only 5.5% of *P. multocida* isolates from pigs were resistant to penicillin and 4.8% were resistant to ampicillin [27].

Chloramphenicol may cause adverse effects in humans and it is only recommended when safer antimicrobials cannot be used. Though previous authors found a high level of susceptibility (< 3% of isolates were resistant) to this drug [18, 24, 25, 32], in our study 17.3% of *P. multocida* type B isolates from swine were resistant to it. In agreement with our results, a study carried out in Korea indicated that 18.5% of type A or D *P. multocida* isolates from pigs were resistant to florfenicol [27]. This drug, a phenicol used widely in veterinary medicine to treat pneumonia caused by *P. multocida,* belongs to the same antimicrobial group as chloramphenicol*.* Indeed, previous authors have found florfenicol to be highly effective [33]. Likewise, gentamicin exhibited moderate activity against the swine isolates we tested in this study (84.5% were susceptible). Similar results were reported by Tang et al. [18] who found 86.3% of their isolates were susceptible to this antibiotic, although other authors have described lower resistance rates (< 3.3%) to gentamicin in *P. multocida* isolates [24, 26, 27, 32].

Enrofloxacin and ceftiofur seemed to have the highest in vitro activity against the isolates we tested: only 4 of 58 (6.9%) of the *P. multocida* type B isolates from pigs that we tested were not susceptible to enrofloxacin, and all of them were vulnerable to ceftiofur. This finding is in accordance with a situation that has remained constant in Spain since 1987 [24, 25]. Additionally, research carried out in Europe between 2009 and 2012 with total of 152 *P. multocida* isolates from pigs failed to find any that were resistant to ceftiofur or enrofloxacin [28]. Moreover, high susceptibility rates to these drugs have been reported for *P. multocida* isolates from pigs in China or Korea [18, 27]. However, Brazilian authors reported that in both cases, 22.5% of their swine isolates were resistant to enrofloxacin and ceftiofur [26]. Although ceftiofur, enrofloxacin, and gentamicin are highly effective antibiotics against *P. multocida*, according to the OIE and WHO, cephalosporins (3rd, 4th, and 5th generation), macrolides, and aminoglycosides are critically important antibiotics for human medicine and animal health [22, 23]. Therefore, their use is only recommended as an emergency treatment in the early stages of HS, until the susceptibility test results are known.

We were unable to determine the resistance rates of erythromycin and neomycin in this study because no break points for *P. multocida* have been defined by the CLSI for these drugs. The MIC values for erythromycin for the isolates tested from both hosts in this research were between 0.125 and ≥ 16 μg/mL, and in most cases (42/76) were ≥ 16 μg/mL. Tang et al. [18] used 8 μg/mL as the break point for this macrolide; thus, applying this break point to the type B *P. multocida* isolates we used in this current study would have meant that 58.6% of those from pigs and 44.4% from cattle would have been classified as resistant to erythromycin. Some authors have reported moderate susceptibility to this drug [25, 32] and in other research, 40% of *P. multocida* isolates from pigs were resistant to erythromycin [26]. Others tested the susceptibility of 25 swine *P. multocida* type B isolates against tildipirosin and found that this macrolide inhibited the growth of more than 80% of the isolates [34]. In our study, the MIC_50_ and MIC_90_ values for erythromycin (both ≥16 μg/mL) were higher than those described by previous authors at 2 μg/mL and 4 μg/mL for MIC_50_ and MIC_90,_ respectively [18, 24]. The MIC values for neomycin in this current study were between 1 and ≥ 16 μg/mL for swine isolates and 0.006 and 8 μg/mL for bovine isolates, and the MIC_50_ and MIC_90_ of the swine isolates were similar to those described previously in type A or D *P. multocida* isolates from swine [18, 24, 27].

MDR has increased over time, partly because of the widespread use of antimicrobials in human and veterinary medicine and antibiotic additives used in animal feed [18]. Additionally, the horizontal transfer of genes through different bacterial species promotes MDR development [19]. Lizarazo et al. [24] reported that in Spain, 7.93% of isolates were resistant to at least four antimicrobials between 1987 and 1988 and that this had risen to 61.9% of those studied between 2003 and 2004. In China, others have described an increase in the prevalence of isolates resistant to more than five antibiotics (from 47.8% in 2003 to 97.1% in 2007) or more than seven antibiotics (from 16.2% in 2003 to 62.8% in 2007) [18]. Of note, there was a sharp increase in the prevalence of MDR among capsular types A and D *P. multocida* isolates from pigs. In this current study on capsular type B isolates, while no MDR could be found in isolates from cattle, 25.86% of those from pigs were resistant to three or more antibiotic classes.

Perhaps the high rate of resistance and number of MDR isolates found for swine in our study can be explained by the more intensive use of antibiotics in the pig industry compared to the cattle industry in Spain. The official European JIACRA (joint inter-agency antimicrobial consumption and resistance analysis) report indicates that while antibiotic consumption for cattle—expressed in mg/PCU (population correction unit)—in Spain was around 900 mg/PCU between 2010 and 2016, in pigs it was more than 3500 mg/PCU. This report also states that 58.51% of ß-lactams were used in the pig industry while 28.43% were used in ruminants; in the case of tetracyclines, 58% were used in the pig industry and only 14% in ruminants [21]. In agreement with other studies which highlighted tetracycline and lincomycin as the antimicrobials most commonly involved in resistance patterns [18, 27], lincomycin, penicillin, and tetracycline were most frequently included in the resistance patterns we observed here.

## Conclusions

In this study we report the lower in vitro resistance rates of *P. multocida* type B isolates from cattle to several antibiotics, as well as a lower prevalence of MDR in bovine isolates, compared to those isolated from pigs. This can be explained by the extensive use of antibiotics in the pig industry in Spain. Lincosamides showed poor activity against all types of *P. multocida* isolates and therefore, is not recommended for treating diseases in cattle or pigs caused by this pathogen. Based on the susceptibility of *P. multocida* type B isolates in this study, the use of ceftiofur, enrofloxacin, or gentamicin is preferable as an emergency treatment in the early stages of HS until susceptibility test results are known, and thereafter, therapeutic or metaphylactic treatments with tetracycline, oxytetracycline, penicillin, or chloramphenicol should be prioritized accordingly. The rapid increase in *P. multocida* isolate resistance against important groups of antimicrobial agents indicates that more attention should be paid to disease prevention and the responsible use of antibiotics, especially those which are important to human health, in order to limit the emergence and spread of antibiotic-resistant bacteria in humans and animals.

## Methods

### Bacterial isolates and identification

A total of 76 *P. multocida* type B isolates (58 porcine and 18 bovine) were evaluated in this study. The isolates were collected between 2009 and 2015 from different outbreaks of acute septicemic pasteurellosis that caused a high mortality rate in pigs and cattle located in 11 different extensively reared systems. Only isolates identified as *P. multocida* type B were assessed in the study. For this purpose, samples were plated on blood agar (Oxoid®) supplemented with 5% defibrinated sheep blood and incubated at 37 °C for 24 h under microaerobic conditions. The initial identification was done based on phenotypic and biochemical properties and was further confirmed by detection of the *kmt1* gene in a species-specific PCR assay [35]. The capsular type was determined using the PCR assay protocol described by Townsend et al. [36], and the biovar was assessed based on the production of the enzyme ornithine decarboxylase, urease activity, and fermentation of seven different carbohydrates [37, 38].

### Antimicrobial susceptibility evaluation

All *P. multocida* type B isolates were tested for their antimicrobial susceptibility based on their MIC. *Staphylococcus aureus* (ATCC® 29213 [39]; was used as a quality-control strain, and its MIC value ranges are shown in Table 6.

**Table 6:** MIC value ranges for the ATCC® 29,213 *Staphylococcus aureus* quality control strain

Antibiotic	Acceptable MIC value range (μg/mL)
**Erythromycin**	0.25–1
**Lincomycin**	ND
**Neomycin**	ND
**Penicillin**	0.25–2
**Oxytetracycline**	0.12–1^a^
**Tetracycline**	0.12–1
**Gentamicin**	0.12–1
**Enrofloxacin**	0.03–12
**Ceftiofur**	0.25–1
**Chloramphenicol**	2–16

^a^ Reference value for tetracycline. *ND* non-determined value according to the Clinical and Laboratory Standards Institute (CLSI; 2013)

The assays were carried out according to the CLSI VET01-A4 performance standards [39]. This quantitative, in vitro method tests susceptibility to antimicrobials at different dilutions in microdilution plates inoculated by adding 100 μl of each isolate. In this study we tested 10 antimicrobial agents (Vetranal, Sigma-Aldrich®): erythromycin, lincomycin, neomycin, penicillin, oxytetracycline, tetracycline, gentamicin, enrofloxacin, ceftiofur, and chloramphenicol. These drugs are widely used by field veterinarians in pigs and cattle and were recommended on the technical hemorrhagic septicemia card provided by the OIE [40].

The inoculum from each isolate was prepared from colonies that had been plated on blood agar; isolates with a spectrophotometric absorbance at 625 nm of 0.08 to 0.13 were used [39]. The inoculum was diluted on a microplate panel and these were fixed with adhesive seals and incubated at 35 °C ± 2 °C for 12 ± 2 h. The MIC was defined as the first dilution at which no visible growth of the isolate was detected in the presence of the antimicrobial being tested. The MICs for lincomycin, penicillin, oxytetracycline, tetracycline, gentamicin, enrofloxacin, and ceftiofur were interpreted using the break points provided by the CLSI guidelines [41]. No break point values for *P. multocida* were available for erythromycin or neomycin (Tables 2 and 3). *P. multocida* isolates resistant to three or more different antimicrobial classes were defined as MDR isolates [42]. The MIC_50_ and MIC_90_ were the MICs that inhibited the growth of 50 or 90% of the isolates, respectively.

### Statistical analysis

Statistical testing was performed with the SPSS software package, version 25.0 (IBM Corp., Armonk, NY). The resistance rates of isolates from both hosts studied as well as MDR isolates were compared using *X*^2^ tests. In any category where *n* ≤ 5, Fisher’s exact test was used. In all cases, *p* values of ≤0.05 were considered statistically significant.

## Data Availability

All data supporting these research findings are included within the manuscript. The databases (without personally identifiable information) are available from the corresponding author upon request.

## Abbreviations

AGISAR: Advisory group on integrated surveillance of antimicrobial resistance
CLSI: Clinical and laboratory standards institute
HS: Hemorrhagic septicemia
MDR: Multidrug resistant
MIC: Minimum inhibitory concentration
MIC_50_: MIC that inhibited the growth of 50% of the isolates
MIC_90_: MIC that inhibited the growth of 90% of the isolates
OIE: World organization for animal health
WHO: World health organization.
